# Familial Xp11.22 microdeletion including *SHROOM4* and *CLCN5* is associated with intellectual disability, short stature, microcephaly and Dent disease: a case report

**DOI:** 10.1186/s12920-018-0471-6

**Published:** 2019-01-10

**Authors:** Magdalena Danyel, Eun Kyung Suk, Vera Raile, Jutta Gellermann, Alexej Knaus, Denise Horn

**Affiliations:** 1 Institute of Medical Genetics and Human Genetics, Charité – Universitätsmedizin Berlin, corporate member of Freie Universität Berlin, Humboldt-Universität zu Berlin, and Berlin Institute of Health, Berlin, Germany; 2 Praxis für Humangenetik-Friedrichstrasse, Berlin, Germany; 3 Department of Pediatric Neurology, Sozialpädiatrisches Zentrum (SPZ), Center for Chronically Sick Children, Charité – Universitätsmedizin Berlin, Germany, corporate member of Freie Universität Berlin, Humboldt-Universität zu Berlin, and Berlin Institute of Health, Berlin, Germany; 4 Department of Pediatric Nephrology, Charité – Universitätsmedizin Berlin, corporate member of Freie Universität Berlin, Humboldt-Universität zu Berlin, and Berlin Institute of Health, Berlin, Germany; 5 Institute for Genomic Statistics and Bioinformatics, University Hospital Bonn, Berlin, Germany

**Keywords:** Dent disease, *CLCN5*, *SHROOM4*

## Abstract

**Background:**

Two interstitial microdeletions Xp11.22 including the *CLCN5* and *SHROOM4* genes were recently reported in a male individual affected with Dent disease, short stature, psychomotor delay and minor facial anomalies. Dent disease, characterized by a specific renal phenotype, is caused by truncating mutations of *CLCN5* in the majority of affected cases.

**Case presentation:**

Here, we present clinical and molecular findings in a male patient with clinical signs of Dent disease, developmental delay, short stature, microcephaly, and facial dysmorphism. Using molecular karyotyping we identified a hemizygous interstitial microdeletion Xp11.23p.11.22 of about 700 kb, which was inherited from his asymptomatic mother. Among the six deleted genes is *CLCN5,* which explains the renal phenotype in our patient. *SHROOM4,* which is partially deleted in this patient, is involved in neuronal development and was shown to be associated with X-linked intellectual disability. This is a candidate gene, the loss of which is thought to be associated with his further clinical manifestations.

To rule out mutations in other genes related to intellectual disability, whole exome sequencing was performed. No other pathogenic variants that could explain the phenotypic features, were found.

**Conclusion:**

We compared the clinical findings of the patient presented here with the reported case with an Xp11.22 microdeletion including *CLCN5* and *SHROOM4* and re-defined the phenotypic spectrum associated with this microdeletion.

**Electronic supplementary material:**

The online version of this article (10.1186/s12920-018-0471-6) contains supplementary material, which is available to authorized users.

## Background

Dent disease is a well-known X-linked renal phenotype which is characterized by progressive proximal renal tubulopathy with low molecular weight proteinuria, nephrocalcinosis and hypercalciuria [1]. The condition varies in degrees of severity and is present in clinically related forms such as X-linked nephrolithiasis or hypercalciuric nephrolithiasis. Female carriers are usually asymptomatic, but show mild proteinuria and hypercalciuria occurs in about 50%. *CLCN5* mutations which are scattered throughout the coding region were detected in these patients [2]. The majority of these mutations are predicted to result in a truncated protein. In about 15% of patients, *OCRL1* mutations are responsible for Dent disease [3].

*SHROOM4,* also known as *KIAA1202* was shown to be associated with X-linked mental retardation (XLMR) in several unrelated individuals [4]. Two unrelated females with mild/moderate intellectual disabilities were found to have balanced X;autosome translocations with Xp11.2 breakpoints which disrupt *SHROOM4*. Affected individuals of another family with a missense variant in this gene presented with severe intellectual disability, delayed or no speech development, seizures, and hyperactivity [4]. Further molecular analyses showed expression of the human *SHROOM4* in adult and fetal brain structures, and indicated its role in the function of specific neuronal population of cells by encoding a protein involved in cytoskeletal architecture [5]. Using array-based comparative genomic hybridization (array CGH) and a whole exome sequencing approach, two maternally inherited X-linked variants in *SHROOM4* and in *ZFX* were identified in a patient with an idiopathic neurodevelopmental phenotype that clinically overlap with Rett syndrome [6].

Recently, two maternally inherited interstitial microdeletions Xp11.22 including the *CLCN5* and *SHROOM4* genes were identified in a male patient [7]. This was the first report of this specific microdeletion in a patient presented with Dent disease, short stature and severe psychomotor delay. To address the question of the full spectrum of clinical variability, we analyzed a further patient with a maternally inherited microdeletion Xp11.22 including *CLCN5* and *SHROOM4*.

## Case presentation

The patient was a 4-year-old boy, the first child of non-consanguineous parents of Croatian origin. The family history was unremarkable. The pregnancy was complicated by polyhydramnios. The boy was delivered by caesarean section at 39 weeks of gestation with a length of 56 cm (+ 1.8 SD), weight of 3640 g (+ 0.4 SD) and occipitofrontal head circumference (OFC) of 35 cm (mean). The Apgar scores were 10, 10, and 10 at 1, 5, and 10 min, respectively, and the umbilical arterial cord pH was normal with 7.30. After birth, physical examination showed a maldescensus of testes which was surgically corrected in the first year of life.

**Fig. 1:**
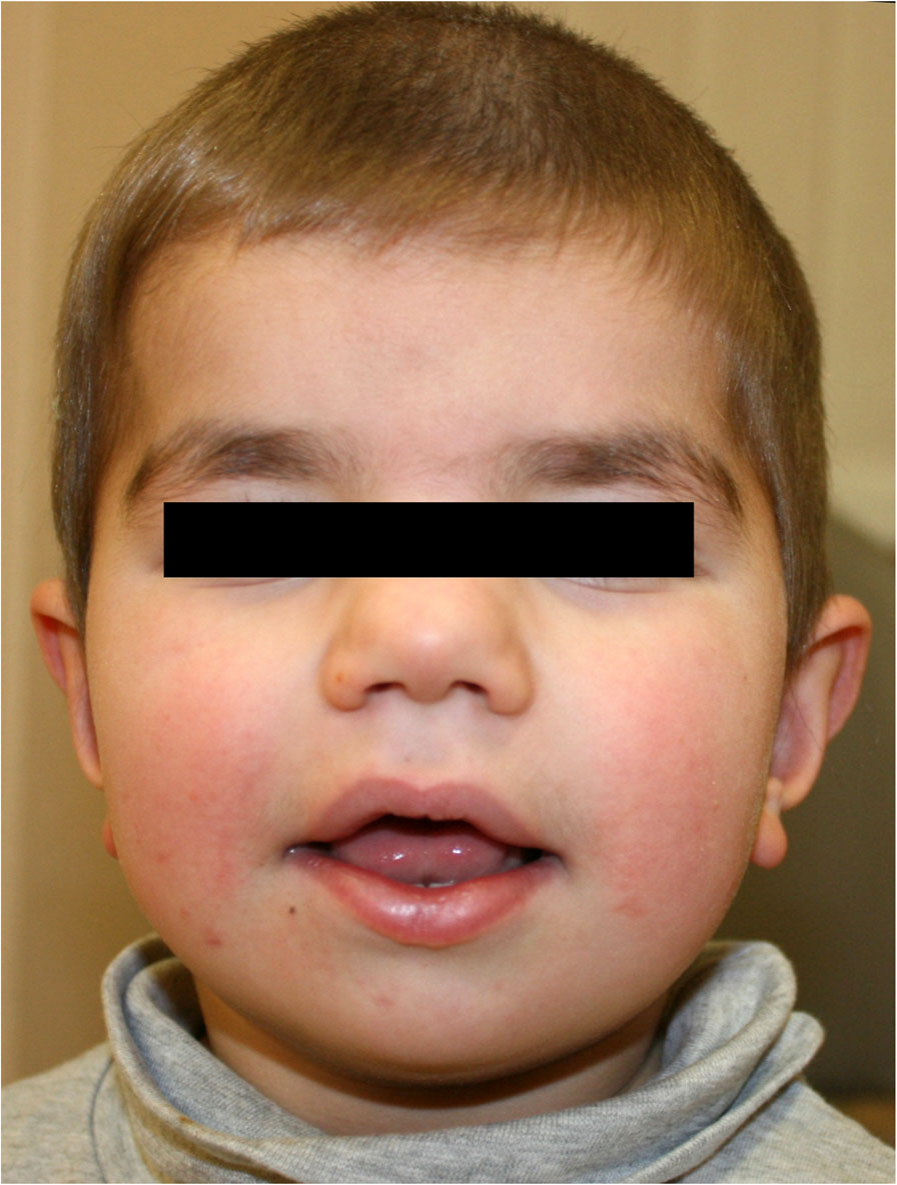
Facial aspect of the patient with Xp11.22 microdeletion at the age of 4 years, showing laterally broad eyebrows, epicanthus, a short nose with a broad nasal bridge, a wide mouth with full lips, and uplifted earlobes

He started walking at 14 months. His speech development was delayed. At the age of 2 years and 6 months his speech and language development was assessed as that of a child of one year and 6 months. At 33 months, he was able to comprehend simple questions and commands and could speak two word sentences. At this time, his length was 84 cm (− 3 SD), his weight was 11.7 kg (− 2 SD) and his OFC was 45.5 cm (− 3.8 SD). His facial features included thick and laterally broad eyebrows, wide set eyes, a short nose with a broad nasal bridge and nasal tip, epicanthus, a wide mouth with full lips, uplifted earlobes, low posterior hairline and dorsal hypertrichosis (Fig. 1). His fingers were short with bilateral clinodactyly V. His toes were also short. At the age of 3 years, septic arthritis of the left hip occurred requiring antibiotic drug treatment. During this treatment a closure of the left femoral artery occurred which required surgical recanalization. X-ray examination of the hand showed that, according to Greulich and Pyle, his skeletal age of 1.5 years, at the chronological age of 2.5 years, was retarded. Furthermore, re-infected cancellous bone markings, cup-shaped metaphyses of the distal radius and ulna on the left with incipient sclerosis, only slight blurring and narrow cortex of the phalanges were detected, indicating compensated hypophosphatemic rickets.

At that time, abdominal ultrasound examination indicated that bilateral nephrocalcinosis was at stage IIa. Further diagnosis revealed partially generalized hyperaminoaciduria as seen in Fanconi syndrome, macroalbuminuria and hypophosphatemia (Additional file 1: Table S1). In addition, there was pronounced tubular proteinuria. The findings were compatible with tubulo-intestinal nephropathy. He was treated with an angiotensin-converting-enzyme inhibitor, he was supplemented with phosphate and vitamin D.

In the neuropediatric examination at the age of 4 4/12 years, the patient showed mild global developmental delay, including speech delay and mild intellectual disability. He was able to speak fluently in short sentences, but grammatical structure and articulation were reduced. Speech comprehension was good. Fine motor function was slightly reduced. He showed adequate social contact with good face-to-face-interaction and no sign of an autistic spectrum disorder. At this time, he received speech therapy and occupational therapy.. He achieved sphincter control during the day at the age of 3 3/12 years, but had not achieved sphincter control during the night at the age of 4 4/12 years. Echocardiography showed no discernible valve defects. At the age of 4 4/12 years, his length was 92 cm (− 3.2 SD), his weight was 14.0 kg (− 1.81 SD), his OFC was 46.5 cm (− 3.8 SD) and his body-mass-index was 16.5 kg/m2 (+ 0.71 SD). His mother was tall (180 cm), had nystagmus but was otherwise healthy. Low-molecular proteinuria was found in her urine analysis.

The results of cytogenetic analysis of the index patient were normal. A microdeletion 22q11.2 was excluded by Fluorescence in situ Hybridization (FISH) analysis.

Standard cytogenetic analysis with a high resolution 550 GTG-banding was performed according to standard procedures, using a lithium-heparin peripheral blood sample from the patient and his mother.

High molecular weight genomic DNA from the patient was isolated from an EDTA peripheral blood sample (Qiagen, QIAamp DNA Blood Midi Kit) and processed according to manufacturer’s protocol for the CytoScan 750 K assay. The genomic DNA was cut by restriction digest, adaptor-ligated and PCR-amplified. Purified amplicons were fragmented by DNAse digest, end-labeled with biotin and hybridized onto an array. After washing and staining, the array was scanned using the Affymetrix GCS3000Dx V2 system. The DNA sample from this patient was analyzed with the Affymetrix microarray platform (GeneChip® System 3000Dx v.2). Primary Image Data were analyzed using the Chromosome Analysis Suite (ChAS) software version 3.1.1.27 with parameter settings allowing the detection of copy number changes of at least 50 kb and smaller and detection of contiguous regions of homozygosity (ROH) > 5 Mb. The copy number changes were analyzed by using multiple clinically relevant databases (e.g. DECIPHER, OMIM, DGV, UCSC, PubMed) and our own patient database. Interpretation and classification in terms of pathogenicity were carried out according to the American College of Medical Genetics guidelines [8] [9].

To clarify whether *SHROOM4* is also affected by the microdeletion Xp11.23p11.22, we performed real-time quantitative PCR (qPCR) for the patient and his mother, measuring two amplicons covering the *SHROOM4* and the *CLCN5* genes. Genomic DNA from the patient and his mother was extracted from EDTA peripheral blood samples. LNA (locked nucleic acid) probes, contained within the Universal Probe Library, and primers were designed with the Roche Probe Finder Software. Real-time quantification of primer and probe pairs was carried out in comparison to a single-copy gene with efficiency correction using a calibrator normalization to determine the copy number. The qPCR analysis was performed on a Roche LightCycler v2 Instrument. The primer sequences are annotated in Additional file 1: Table S2.

Whole exome sequencing was performed on DNA extracted from blood. Library preparation of 100 ng DNA was performed according to the manufacturer’s protocol using SureSelect Human All Exon V6 (Agilent, Waldbronn Germany). Sequencing was performed on the Illumina HiSeq 2500 platform. Reads were aligned to the reference genome (Human Genome Assembly GRCh37) with Burrows-Wheeler Aligner (BWA) [10] and variants were called according to best practice guidelines [11]. For further analysis, all variants that have been reported in a homozygous state in more than three individuals in the cohorts of the 1kGP project, ExAC, gnomAD, or variants that have been classified as benign in ClinVar, were removed.

Variant analysis was carried out with GeneTalk [12]. Rare and coding, heterozygous, compound heterozygous, homozygous, and hemizygous variants in known ID (intellectual disability) genes were assessed with MutationTaster [13].

**Fig. 2:**
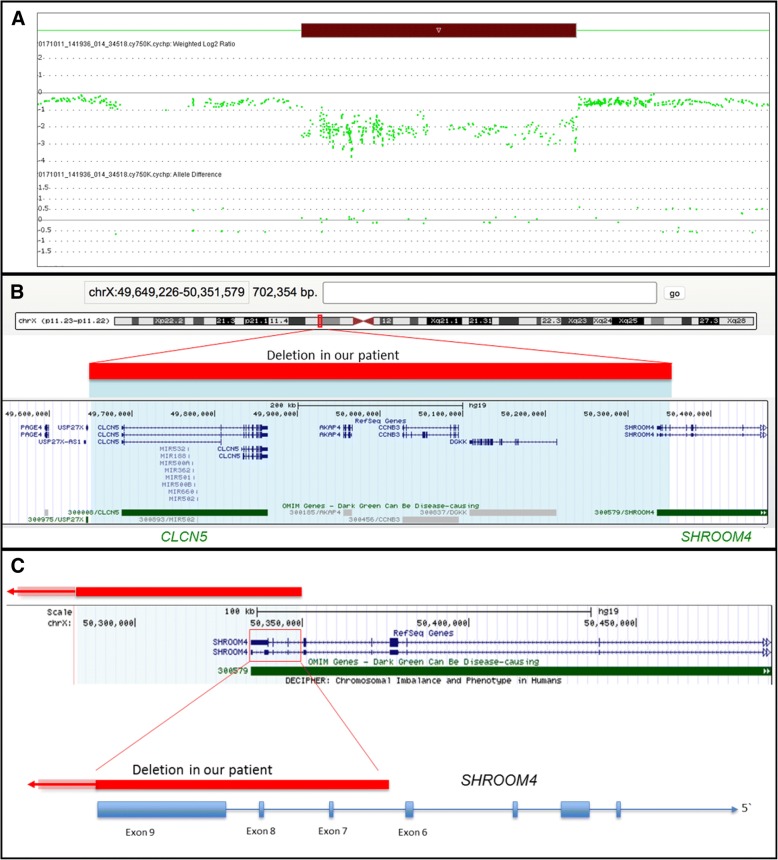
**a**. Deletion in our patient indicated by red bar by ChAS Software. Decreased weighted Log2Ratios and collapsed allele difference tracks point to a hemizygous deletion (copy number 0) spanning genomic position 49,649,22,650,351,579 bp (hg19). **b**. Deletion in our patient (red bar) covering two disease-related genes: *CLCN5* and parts of *SHROOM4* (green bar) and three other genes (*AKAP4, CCNB3, DGKK*). **c**. Deletion in our patient (red bar) encompasses last coding exons of *SHROOM4*

While the standard cytogenetic analysis showed a normal male karyotype in this patient, we identified, by molecular karyotyping, a hemizygous interstitial microdeletion Xp11.23p.11.22 of about 700 kb, according to HG19 arr Xp11.23p.11.22 (49,649,226_50,351,579)× 0 (Fig. 2a).

The genome coordinates chrX: 49649226_50,351,579 in the UCSC human genome 19 indicated that the deletion included *CLCN5,* the last three exons of *SHROOM4* and four additional genes (Fig. 2b and c).

For confirmation, we used target-specific qPCR analysis and validated this microdeletion, including a deletion of *SHROOM4* in the patient and his mother. The coordinates for the minimum and maximum deletion intervals are chrX: 49649226–50,351,579 bp and chrX:. 49648874–50,358,217 bp, respectively.

To rule out mutations in known genes related to intellectual disability, whole exome sequencing was performed. No known or predicted pathogenic mutations, that could explain the phenotypic features, were identified. However, the alignment of reads on chromosome X confirmed the deletion identified by array CGH.

## Discussion and conclusions

This report presents a familial interstitial microdeletion of Xp11.23p.11.22 in a male patient with delayed speech development, mild intellectual disability, growth retardation, microcephaly, facial abnormalities and a renal phenotype with proteinuria, nephrocalcinosis and hypophosphatemia. Maternal transmission of this deletion was confirmed by qPCR tests. Among the six deleted genes, two are associated with diseases: The loss of *CLCN5* could explain his renal phenotype, and *SHROOM4* is a possible candidate gene for at least some of the other clinical manifestations.

*SHROOM4* has been shown to be associated with X-linked mental retardation [4]. Affected males from one family with a missense variant of this gene show severe intellectual disability, bilateral congenital hip luxation, and short stature [14]. Recently, a study of 19 patients with Rett-*like* phenotypes, identified two X-linked variants in *SHROOM4* and *ZFX* in one individual [6]. This patient was a 14-year-old boy, and had severe intellectual disability with microcephaly, peripheral vasomotor disturbances, an attenuated response to pain, and autistic traits. In addition, dyspraxic gait, severe speech delay, eye pointing, and kyphosis were observed. To date, *ZFX* has been shown to be involved in human sex determination and to be associated with retinoschisis and the phenotype of Turner syndrome, and thought to be a candidate gene for non syndromic primary ovarian insufficiency [15, 16]. A link between the *ZFX* variant and the clinical manifestations of the reported patient is not known at present. Therefore this phenotype could be explained by the second X-linked variant in *SHROOM4*.

To date, only one male patient has been reported to carry two neighboring Xp11.22 microdeletions, including *CLCN5* and *SHROOM4* [7]*.* The microdeletions were detected by array CGH (180 K) and one had a size of 148 kb and included the *CLCN5* gene and a second gene. The other microdeletion from the described patient had a size of 2.6 Mb and contained 23 genes including *SHROOM4.* This 4 ½ year-old patient exhibited severe psychomotor delay, especially retarded speech development as well as short stature, facial dysmorphism, including a large forehead, and renal tubulopathy. Furthermore, he developed hydrocephalus due to stenosis of the aquaeduct of Sylvius.

Compared to the clinical findings in our patient, there is a clear overlap with regard to speech and developmental delay, short stature and the renal phenotype (Table 1). Mild craniofacial anomalies present in both include laterally broad eyebrows, and a short nose with a broad nasal bridge. The additional findings of hydrocephalus in the reported patient and microcephaly in our patient may be part of the clinical spectrum associated with this microdeletion.

**Table 1:** Comparison of two male patients affected with Xp11.22 microdeletion including *SHROOM4* and *CLCN5*

	Patient reported by *Armanet* et al.	Patient reported here
Xp11.22 microdeletion	+	+
Age	4.5 years	4 years
Speech delay	Severe	+
Global developmental delay	+	mild
Other neurological abnormalities	Hydrocephalus	–
Short stature	+	+
Microcephaly	–	+
Facial dysmorphisms	+	+
Dent disease	+	+

There is a considerable clinical overlap with patients affected by the oculocerebrorenal syndrome, also referred to as Lowe syndrome. The oculocerebrorenal syndrome caused by *OCRL1* mutations is mainly characterized by the triad congenital cataracts, intellectual disability and renal tubular dysfunction. However, the ocular manifestations can be missing or can occur later in life, in the second or third decade. Therefore, the oculocerebrorenal syndrome represents an important differential diagnosis of the condition described here [17].

The known Xp11.23p11.2 microduplication can be divided into the 4.5 Mb recurrent duplication which includes the *SHROOM4* gene, and atypical microduplications of different sizes. Within the recurrent 4.5 Mb microduplication the *SHROOM4* gene is considered to be one of the candidate genes for intellectual disability. In addition to intellectual disability carriers of this microduplication are affected by seizures and early onset of puberty in female patients [18]. In summary, our study shows that haploinsufficiency of *CLCN5* and *SHROOM4* is associated with tubulopathy and a syndromic form of ID with microcephaly and facial dysmorphisms, as well as short stature. These findings further expand the clinical spectrum of patients with Xp11.22 deletions.

## Additional file

Additional file 1: **Table S1.** Quantitative findings on renal function. Abnormal laboratory parameters are bolded. All parameters were assessed during therapy with Ramiril 1.25 mg. **Table S2.** Primer Sequences and localisation of qPCR amplicons. (DOCX 16 kb)

## Abbreviations

Array CGH: Array-based Comparative Genomic Hybridization
Bp: Base pair
BWA: Burrows-Wheeler Aligner
FISH: Fluorescence in situ Hybridization
GRCh37, HG19: Human Genome Assembly GRCh37
ID: Intellectual disability
Kb: Kilo-base pair
LNA: Locked nucleic acid
OFC: Occipitofrontal circumference
qPCR: Real-time quantitative PCR
SD: Standard deviation
