# Supplementary material for: Familial Xp11.22 microdeletion including SHROOM4 and CLCN5 is associated with intellectual disability, short stature, microcephaly and Dent disease: a case report
Source: BMC Med Genomics. 2019 Jan 10;12:6. doi: 10.1186/s12920-018-0471-6 (PMC6327553; doi:10.1186/s12920-018-0471-6)
Supplement: Supplementary file 1 — Table S1. Quantitative findings on renal function. Abnormal laboratory parameters are bolded. All parameters were assessed during therapy with Ramiril 1.25 mg. Table S2. Primer Sequences and localisation of qPCR amplicons. (DOCX 16 kb) [file 12920_2018_471_MOESM1_ESM.docx]

**Additional file 1**

**Table S1.** **Quantitative findings on renal function.** Abnormal laboratory parameters are bolded. All parameters were assessed during therapy with Ramiril 1.25 mg.

| **Laboratory parameter** | **Result** | **Unit** | **Reference range** |
| --- | --- | --- | --- |
| Total protein in serum | 7.5 | g/dl | 6.0-8.0 |
| Albumin in serum | 46.9 | g/l | 38.0-58.0 |
| Urea in serum | 42.7 | mg/dl | 10.0-48.0 |
| Creatinine in serum | 0.35 | mg/dl | <0.49 |
| Calcium in serum | **2.66** | mmol/l | 2.10-2.60 |
| Calcium in urine | **2.97** | mmol/l |  |
| Calcium/Creatinine ratio | **2.84** | mmol/mmol | <1.13 |
| Inorganic phosphate in serum | 1.11 | mmol/l | 0.97-1.94 |
| Sodium in serum | 135 | mmol/l | 132-145 |
| Potassium in serum | 4.0 | mmol/l | 3.1-5.1 |
| Creatinine in urine | **20** | mg/dl | 40-278 |
| Protein in urine | **693** | mg/dl | <100 |
| Albumin in urine | **180.2** | mg/dl |  |
| Immunglobulin G in urine | **30.50** | mg/dl | <5.30 |
| Alpha 1 microglobulin in urine | **127.0** | mg/dl | <4.8 |

**Table S2.** Primer Sequences and localisation of qPCR amplicons

| **chromosome** | **gene** | **position** | **probe** | **forward primer** | **reverse primer** |
| --- | --- | --- | --- | --- | --- |
| Xp11.23p11.22 | *CLCN5* | 49851342-49851415 | UPL | ctgtttaatgactgtggcctct | cctttgcttgtgttgaaacg |
| Xp11.23p11.22 | *SHROOM4* | 50339429-50339489 | UPL | agctgcagcaaggtgtgat | acttgcccttggcaactg |
